# Inhibitory effects of a selective prostaglandin E2 receptor antagonist RQ-15986 on inflammation-related colon tumorigenesis in *APC*-mutant rats

**DOI:** 10.1371/journal.pone.0251942

**Published:** 2021-05-18

**Authors:** Yohei Shirakami, Takayuki Nakanishi, Noritaka Ozawa, Takayasu Ideta, Takahiro Kochi, Masaya Kubota, Hiroyasu Sakai, Takashi Ibuka, Takuji Tanaka, Masahito Shimizu

**Affiliations:** 1 Departments of Gastroenterology/Internal Medicine, Gifu University Graduate School of Medicine, Gifu, Japan; 2 Department of Pathological Diagnosis, Gifu Municipal Hospital, Gifu, Japan; Kurume University School of Medicine, JAPAN

## Abstract

Prostaglandin E2 receptor EP4 is involved in inflammation and related tumorigenesis in the colorectum. This study aimed to investigate the chemopreventive ability of RQ-15986, a selective EP4 antagonist, in colitis-related colorectal tumorigenesis. Male Kyoto APC delta rats, which have APC mutations, were treated with azoxymethane and dextran sulfate sodium and subsequently administered RQ-15986 for eight weeks. At the end of the experiment, the development of colorectal tumor was significantly inhibited in the RQ-15986-treated group. The cell proliferation of the crypts and tumors in the colorectum was decreased following RQ-15986 treatment. RQ-15986 also suppressed the expression of pro-inflammatory cytokines, including tumor necrosis factor-α, interleukin-6, interleukin-18, and monocyte chemotactic protein-1, in the colon mucosa. In addition, the expression levels of indoleamine 2,3-dioxygenase, which is involved in immune tolerance, were decreased in the colorectal epithelium and tumors of the RQ-15986-treated group. These findings indicate that RQ-15986 inhibits colitis-associated colorectal tumorigenesis by attenuating inflammation, suppressing cell proliferation, and modulating the expression of indoleamine 2,3-dioxygenase. Targeting prostaglandin E2/EP4 signaling might be a useful strategy for chemoprevention of inflammation-related colorectal cancer.

## Introduction

Colorectal cancer (CRC) is one of the most serious health concerns owing to its high morbidity and mortality. Because the prevalence of CRC has increased gradually worldwide [1], there is a critical need to develop more effective strategies to prevent and treat this malignancy. Chronic inflammation is widely accepted as a major risk factor for CRC. Patients with inflammatory bowel diseases, such as ulcerative colitis and Crohn’s disease, are at high risk of CRC [2]. Several inflammatory cytokines, including tumor necrosis factor (TNF)-α and interleukin (IL)-6, are involved in the development of colitis-associated cancer [3]. Conversely, it has also been reported that attenuation of inflammation is effective for preventing colitis-related CRC [4].

Prostaglandins are inflammatory mediators generated from arachidonic acids by the enzyme cyclooxygenase-2 (COX-2). Among the prostaglandins, prostaglandin E2 (PGE2) is a key COX-2 product that regulates various inflammatory processes [5]. PGE2 exerts its pleiotropic effects by binding to four different PGE2-sensitive E-type prostanoid (EP) receptors, EP1 to EP4 [5]. Especially, PGE2/EP4 signaling is significantly involved in colorectal inflammation. PGE2/EP4 signaling is suppressed in both ulcerative colitis patients and mice with dextran sulfate sodium (DSS)-induced experimental colitis [6], but supplementation with an EP4-selective agonist significantly improves DSS-induced colitis in mice [7].

In addition to colorectal inflammation, PGE2/EP4 signaling is profoundly associated with colorectal tumorigenesis [8]. In human samples, the levels of PGE2 are significantly elevated in CRC tissues compared with those in the normal mucosa [9]. PGE2 promotes the proliferation, growth, and migration of CRC cells [10]. Furthermore, PGE2/EP4 signaling plays critical roles in stem cell expansion, metastasis, and tumor microenvironment of CRC [10,11]. For instance, the expression of indoleamine 2,3-dioxygenase (IDO), an enzyme that plays a role in induction of immune tolerance, is regulated by PGE2/EP4 signaling [12,13]. Consequently, targeting PGE2/EP4 signaling can be a promising approach for the prevention and treatment of CRC [14,15].

RQ-15986, a selective EP4 antagonist that was originally reported to improve signs and symptoms of osteoarthritis and rheumatoid arthritis [16], is expected to attenuate inflammation. Recent experimental studies have showed the evidence that RQ-15986 exerts anti-tumor effects against breast cancer cells [17,18]. The agent was effective in suppressing tumor growth and tumor-associated angiogenesis of breast cancer cells induced by COX-2/vascular endothelial growth factor pathway [17]. Therefore, we expected that RQ-15986 might suppress inflammation-related colorectal tumorigenesis by attenuating chronic inflammation in the bowel.

The purpose of this study was to clarify the potential chemopreventive ability of a selective EP4 antagonist in colitis-related colorectal tumorigenesis. For this purpose, we used a colitis-related CRC model of Kyoto adenomatous polyposis coli (APC) delta (KAD) rats with an Apc mutation, which mimics human inflammation-related colon carcinogenesis well [19,20]. In the present study, we examined whether RQ-15986 administration prevents the development of tumors in the inflamed colorectum by suppressing the expression of inflammatory cytokines and IDO.

## Materials and methods

### Chemicals and animals

**Fig 1:**
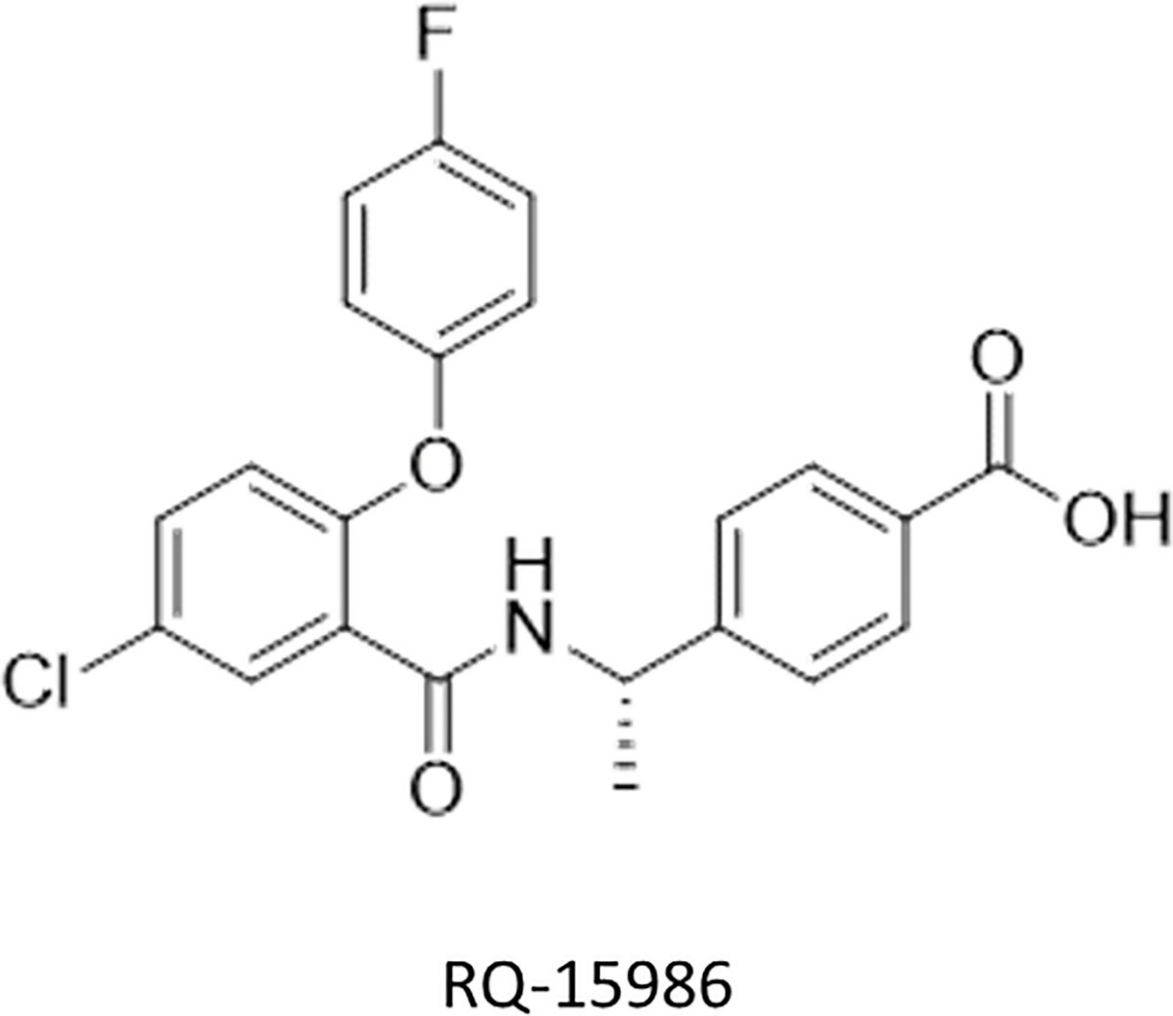
Chemical structure of RQ-15986.

Azoxymethane (AOM) was obtained from Sigma Chemical Co. (St. Louis, MO, USA). DSS was purchased from MP Biomedicals, LLC (Aurora, OH, USA). RQ-15986 (molecular weight 413.83, Fig 1) was kindly provided by AskAt Inc. (Nagoya, Japan). Male KAD rats (5 weeks of age) were purchased from Charles River Japan Inc. (Tokyo, Japan). The mice were maintained at the Gifu University Life Science Research Center according to the Institutional Animal Care Guidelines. The Institutional Animal Care and Use Committee of Gifu University approved all the experimental protocols and procedures of this study (the authorization code 30–7 on 5 April 2018). All mice were housed in plastic cages with free access to drinking water with a pelleted basal diet CRF-1 (Oriental Yeast, Tokyo, Japan).

### Experimental procedure

Details of the experimental procedure for AOM plus DSS-induced colitis-related CRC model of KAD rats have been described previously [19,20] Twenty-seven male KAD rats were divided randomly into experimental and control groups. The rats of group 1 (n = 6) were not administered any treatment and were used as control. The rats of group 2 (n = 6) were orally administered RQ-15986 (10 mg/kg/day) from 10 weeks of age. The rats of groups 3 (n = 7) and 4 (n = 8) were administered a single intraperitoneal injection of AOM (20 mg/kg body weight). From 1 week after the injection of AOM, these rats received DSS (2.0% w/v) in drinking water for 7 days. The rats of group 4 were also administered RQ-15986 concurrently with those of group 2.

All the rats were sacrificed at 18 weeks of age, the size of the visible colorectal tumors was measured, and paraffin-embedded sections of colonic mucosa were prepared using routine procedures for subsequent histopathological and immunohistochemical investigations [20]. To evaluate the development of colorectal tumors sequentially, endoscopic observations were also performed at 12, 14, and 16 weeks of age [20].

### Immunohistochemical analyses

Immunohistochemical staining for proliferating cell nuclear antigen (PCNA) and IDO in the colorectal mucosa was performed using the labeled streptavidin-biotin method (LSAB kit; Dako, Glostrup, Denmark) with primary anti-PCNA antibody (1:100, Santa Cruz Biotechnology, Dallas, TX, USA) and anti-IDO antibody (1:1000; LYFESPAN, Seattle, WA, USA). The positive cell rates of immunohistochemistry for PCNA or IDO were evaluated based on previous methods [20,21].

### RNA extraction and quantitative real-time reverse transcription-PCR analysis

Total RNA was isolated from the scraped colon mucosa of experimental rats using the RNeasy Mini Kit (QIAGEN, Venlo, Netherlands). cDNA was synthesized from 0.2 μg of total RNA using High Capacity cDNA Reverse Transcription Kit (Applied Biosystems, Foster City, CA, USA). Quantitative real-time reverse transcription-PCR (RT-PCR) analysis was performed using LightCycler Nano (Roche Diagnostics, Indianapolis, IN, USA) with LightCycler 480 SYBR Green I Master Mix (Roche Diagnostics). The specific primers used for amplifying forkhead box protein-3 (*Foxp3*), *Ido1*, *Ifng*, *Il6*, *Il18*, monocyte chemotactic protein-1 (*Mcp1*), *Tnfa*, and glyceraldehyde-3-phosphate dehydrogenase (*Gapdh*) genes were designed using the software available at the Roche Universal Probe Library Assay Design Center (https://lifescience.roche.com/global_en/brands/universal-probe-library.html). The sequences for these primers are shown in S1 Table. The expression levels of *Foxp3*, *Ido1*, *Ifng*, *Il6*, *Il18*, *Mcp1*, and *Tnfa* were normalized to the expression level of *Gapdh*.

### Cell lines and cell culture

HT29 and SW837 human CRC cell lines obtained from the JCRB Cell Bank (Osaka, Japan) were maintained in Dulbecco’s modified Eagle’s medium (Sigma) supplemented with 10% fetal bovine serum (Sigma) in an incubator at 37 °C under a humidified atmosphere containing 5% CO2. The cells were preincubated in the absence or presence of RQ-15986 at various concentrations (0.1, 1.0, 5.0, or 10 μM) for 2 h and then stimulated with interferon (IFN)-γ (10 ng/mL) for 24 h. IFN-γ was used for induction of IDO1. Cell extracts were prepared and RT-PCR analysis for *IDO1* was performed. The sequences for the human primers are shown in S1 Table. The expression levels of *IDO1* were normalized to those of *GAPDH*.

### Statistical analysis

All data are expressed as mean ± SD. The differences between the groups were analyzed using two-way ANOVA. For significant differences indicated by ANOVA, Tukey–Kramer multiple comparison test was performed. Fisher’s exact test was used to compare the incidence of intestinal tumors. A value of *P* < 0.05 was considered significant.

## Results

### General observations

The weights of the body, liver, kidneys, and spleen, and the length of the large bowels of the rats of all groups at the end of the study are listed in Table 1. There were no significant differences in body and organ weights among the experimental groups. The length of the large bowel of rats in groups 3 and 4, which received AOM plus DSS, was significantly shorter than that in AOM/DSS-untreated group 1 (*P* < 0.01). During the course of the experiment, administration of RQ-15986 did not cause any clinical symptoms. No pathological alterations that would suggest toxicity of RQ-15986 in the liver, kidney, and spleen of rats were observed (S1 Fig).

**Table 1:** General observations of the experimental rats.

Group no.	Treatment	No. of rats	Body weight (g)	Liver weight (g)	Kidney weight (g)	Spleen weight (g)	Length of large bowel (cm)
1	None	6	331.6 ± 7.4 ^a^	11.3 ± 1.2	2.39 ± 0.1	0.7 ± 0.1	22.8 ± 1.8
2	RQ alone	6	334.8 ± 17.0	10.8 ± 1.2	2.40 ± 0.2	0.6 ± 0.1	22.3 ± 2.4
3	AOM/DSS	7	307.5 ± 37.7	9.8 ± 1.6	2.27 ± 0.3	0.6 ± 0.1	18.4 ± 1.3 ^b^
4	AOM/DSS/RQ	8	320.6 ± 8.6	9.8 ± 0.7	2.20 ± 0.1	0.7 ± 0.0	19.2 ± 1.2 ^b^

^a^ Mean ± SD.

^b^ Significantly different compared to group 1 (*P* < 0.05).

AOM, azoxymethane. DSS, dextran sulfate sodium. RQ, selective EP4 antagonist RQ-15986.

### Effects of RQ-15986 on the development of colorectal tumor in the experimental rats

**Fig 2:**
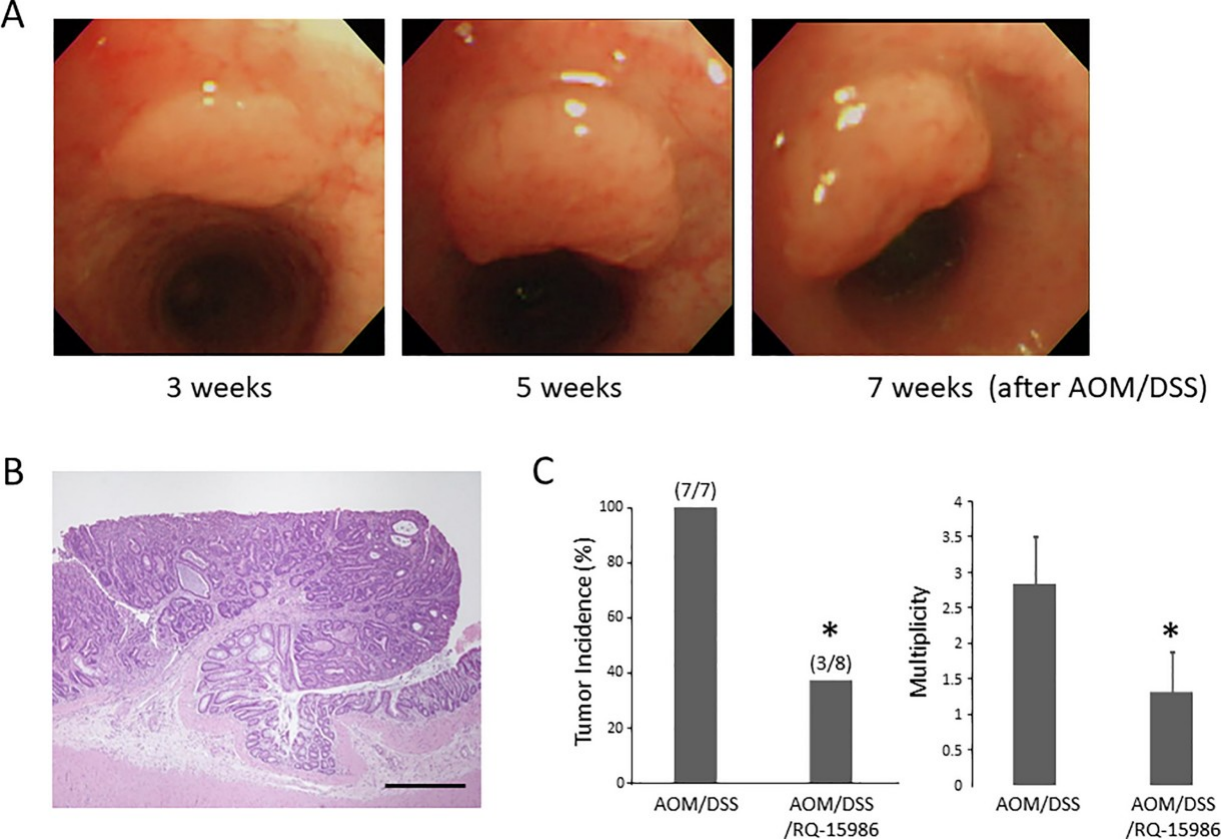
The images of AOM/DSS-induced colon tumors and the effects of RQ15986 on the tumors in KAD rats. Endoscopic (A) and histopathological (B) observations of colon tumors in AOM/DSS-treated KAD rats. (A) Endoscopic images of a colon tumor of 3, 5, and 7 weeks after AOM/DSS administration on the left, center, and right panel, respectively. (B) Hematoxylin and eosin-stained tumor. Scale bar, 500 μm. (C) Incidence and multiplicity of colon tumors. Each column represents the mean ± SD. Asterisk indicates statistically significant difference compared to AOM/DSS group; *P* < 0.05.

Colorectal tumors were observed endoscopically 3 weeks after the end of DSS administration, and they increased over time in the colons of rats that received AOM and DSS (Fig 2A). At sacrifice, colorectal tumors, which were considered as adenomas in histopathological analysis (Fig 2B), were observed only in the colons of AOM/DSS-treated rats. In these rats, RQ-15986 administration significantly decreased the incidence and multiplicity of colorectal tumors (Fig 2C, *P* < 0.05), indicating that RQ-15986 suppressed colitis-related colorectal carcinogenesis.

### Effects of RQ-15986 on cell proliferation in the colonic mucosa and colorectal tumor

**Fig 3:**
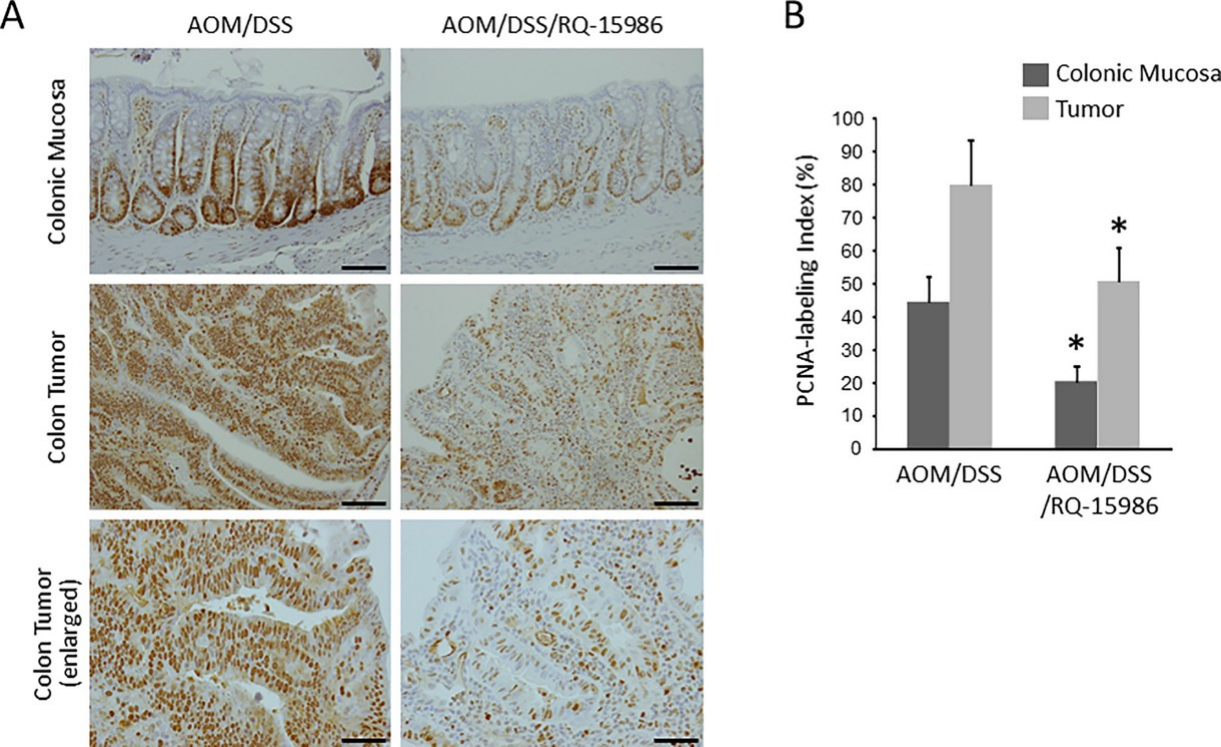
Effects of RQ15986 on cellular proliferation in the colonic mucosa and tumor. (A) Representative images of immunohistochemical staining in the colonic mucosa and tumor of AOM/DSS-treated KAD rats with anti-PCNA antibody. Scale bars, 100 μm. (B) PCNA-labeling index in the colonic mucosa and tumor of AOM/DSS-treated KAD rats. Each column represents the mean ± SD. Asterisk indicates statistically significant difference compared to the index in AOM/DSS group; *P* < 0.05.

The effects of RQ-15986 on cell proliferation in the colonic mucosa and CRC tissues of the experimental rats were evaluated through immunohistochemical staining for PCNA. Both in the non-lesional crypts and CRC tissues, administration of RQ-15986 markedly decreased the PCNA-labeling indices (Fig 3A and 3B). Therefore, RQ-15986 inhibited cell proliferation in the colonic mucosa of AOM/DSS-treated rats, which contributed to the suppression of colorectal tumorigenesis.

### Effects of RQ-15986 on the levels of pro-inflammatory cytokines in the colonic mucosa of experimental rats

**Fig 4:**
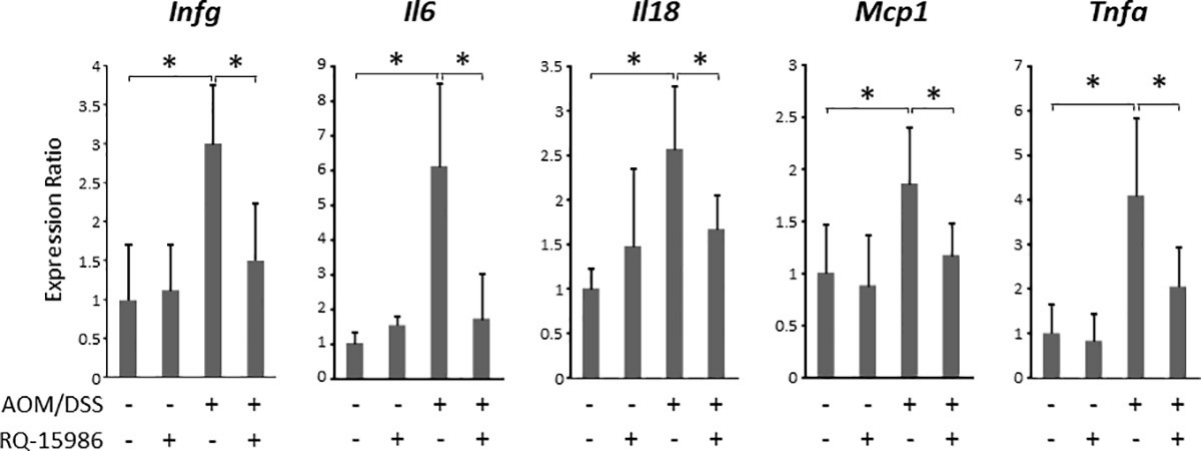
Effects of RQ15986 on the levels of pro-inflammatory cytokines in the colonic mucosa of experimental rats. Total RNA was isolated from colonic mucosa of the experimental rats, and expression levels of pro-inflammatory cytokine mRNA were determined using quantitative real-time RT-PCR with specific primers. Values are expressed as mean ± SD. Asterisk indicates statistically significant difference between the indicated groups; *P* < 0.05.

The effects of RQ-15986 on the mRNA levels of pro-inflammatory cytokines in the colonic mucosa were examined. As shown in Fig 4, the mRNA expression levels of *Ifng*, *Il6*, *Il18*, *Mcp1*, and *Tnfa* were markedly elevated in the colonic mucosa of AOM/DSS-treated rats compared to those in untreated rats. In AOM/DSS-treated rats, RQ-15986 administration significantly decreased the expression levels of these mRNAs. These findings indicate that RQ-15986 attenuated colorectal inflammation associated with tumorigenesis.

### Effects of RQ-15986 on the levels of IDO1 in the colon of experimental rats and CRC cells

**Fig 5:**
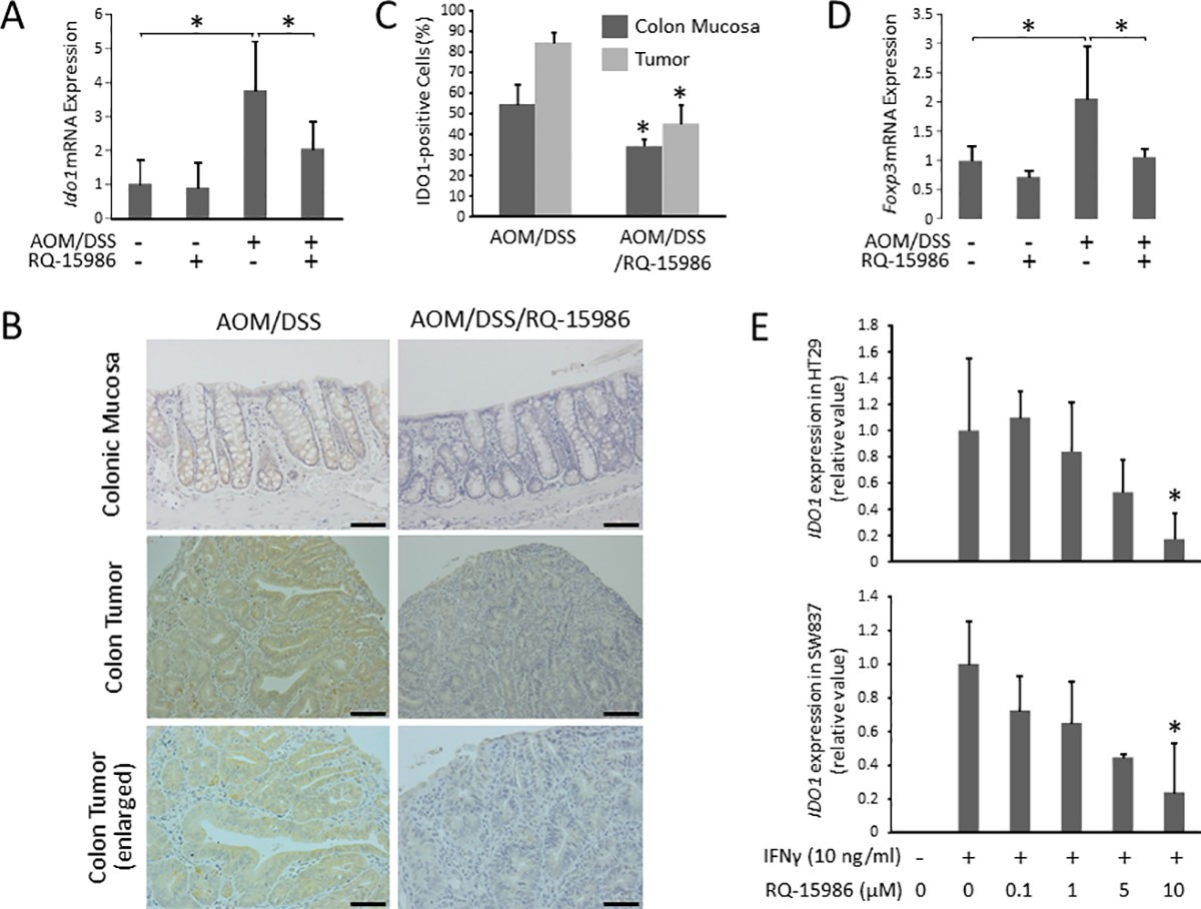
Effects of RQ-15986 on the levels of IDO1 in the colon of experimental rats and CRC cells. (A) Total RNA was isolated from colonic mucosa of the experimental rats, and expression levels of *Ido1* mRNA were determined using quantitative real-time RT-PCR. (B) Representative images of immunohistochemical staining for IDO1 in the colonic mucosa and tumor of AOM/DSS-treated KAD rats. Scale bars, 100 μm. (C) IDO1-positive cells were counted and the positive ratio was calculated. (D) Expression levels of *Foxp3* mRNA were determined using quantitative real-time RT-PCR. Asterisk indicates statistically significant difference compared to the value in AOM/DSS group; *P* < 0.05. (E) HT29 and SW837 human CRC cells were pre-incubated with indicated concentration of RQ-15986 for 2 hours and then stimulated with IFN-γ (10 ng/ml) for 24 hours. Total RNA was isolated from the treated cells and expression levels of *Ido1* mRNA were determined using quantitative real-time RT-PCR. Asterisk indicates statistically significant differences compared to IFN-γ-stimulated/RQ-159860-untreated group; *P* < 0.05. Each column represents the mean ± SD.

We next examined the effects of RQ-15986 on the expression of IDO1 in the colonic mucosa and CRC cells. The expression levels of *Ido1* mRNA in the colonic mucosa were significantly elevated in AOM/DSS-treated rats, but this effect was suppressed upon RQ-15986 administration (Fig 5A). Immunohistochemical analysis also revealed that, in AOM/DSS-treated rats, the expression levels of IDO1 in both CRC tissues and the non-lesional mucosal cells were significantly decreased upon RQ-15986 administration (Fig 5B and 5C). IDO enhances activity of immunosuppressive regulatory T-cell (Treg), which contributes to the escape of pre-malignant and malignant cells from antitumor immune responses [22,23]. In order to investigate the relationship between IDO expression and alterations of immune systems, the levels of Foxp3, one of the most distinctive markers of Treg, were investigated in colonic mucosa of experimental rats. The mRNA levels of *Foxp3* were markedly increased in colonic mucosa of AOM/DSS-treated rats compared to those in non-treated control group; however, the levels were significantly decreased in rats treated by AOM/DSS/RQ15986 as compared to those by AOM/DSS (Fig 5D). In addition, treatment with RQ-15986 (10 μM) suppressed the expression levels of *Ido1* mRNA induced by IFN-γ in HT29 and SW837 human CRC cells (Fig 5E). In addition, treatment with RQ-15986 (10 μM) suppressed the expression levels of *Ido1* mRNA induced by IFN-γ in HT29 and SW837 human CRC cells (Fig 5D). These findings suggest that suppression of IDO1 expression is one of the key mechanisms of RQ-15986 in preventing colitis-related CRC development.

## Discussion

Among the inflammatory mediators, COX-2/PGE2/EP4 signaling plays a critical role in inflammation and tumorigenesis of the colorectum [6–8]. Consequently, inhibition of COX-2/PGE2 using non-steroidal anti-inflammatory drugs (NSAIDs) or COX-2 selective inhibitors could prevent CRC development [24]. Clinical trials have revealed the effectiveness of these agents in the prevention of CRC; however, these studies failed eventually due to severe adverse events such as cardiovascular toxicity [25]. Therefore, as EP4 is a more specific downstream signaling molecule of COX-2/PGE2, targeting EP4 is promising for suppressing inflammation-related CRC development with fewer adverse effects [14,15].

The results of the present study provided the first evidence that the selective EP4 antagonist RQ-15986 significantly suppresses inflammation-related colorectal tumorigenesis without showing toxicity. RQ-15986 administration decreased the expression levels of pro-inflammatory cytokines including TNF-α and IL-6, both of which regulate the development of colitis-associated cancer [3]. RQ-15986 also suppressed the expression levels of MCP-1, an important mediator of tumor growth and immune regulation in the tumor microenvironment of CRC [26]. In addition, the number of PCNA-positive cells in the intestinal epithelium and tumors were also decreased compared to those in control rats, indicating that RQ-15986 suppressed inflammation-related colorectal tumorigenesis through the inhibition of cell proliferation. Further studies clarifying whether and how much RQ-15986 can attenuate colorectal inflammation and subsequently suppress the development of colitis-related CRC in comparison with NSAIDs and COX-2 inhibitors seem important.

Recent studies have revealed that PGE2/EP4 signaling influences the cells in the tumor microenvironment and favors tumor development by exerting immunosuppressive effects [10,27]. The results of the study using *Apc*^Min/+^ mice showed that PGE2/EP4 signaling promotes the escape of tumor cells from antitumor immune response and thus enhances the survival of these cells [28]. PGE2/EP4 signaling is also involved in the upregulation of IDO, which induces immune tolerance [29]. The expression of IDO is regulated by several inflammatory factors, including IFN-γ and COX-2 [30]. In this study, RQ-15986 decreased the expression levels of IDO both in the intestinal mucosa and tumor tissues. RQ-15986 also suppressed the expression of IDO induced by IFN-γ in CRC cells. These results are consistent with a previous report demonstrating that knockdown of EP4 markedly decreased *Ido1* mRNA expression levels in human cancer cells [13]. In addition, administration of an IDO inhibitor has been reported to prevent CRC development in rodent models [21,31]. As well as suppression of cytotoxic T lymphocytes, IDO promotes tumor-related immune tolerance by activating Treg, which are considered as one of the mechanisms for immune suppression in tumor microenvironment [32,33]. In our study, the mRNA levels of *Foxp3*, a marker for Treg, were markedly increased in AOM/DSS-treated rats, but the elevation was inhibited by RQ-15986 administration, which suggests that IDO-induced Treg was inhibited by RQ-15986 administration, contributing to antitumor immune response. Moreover, a clinical study have shown that IDO expression in CRC tissues is related with worse prognosis [34]. The results of the present study together with previous reports above suggest that IDO-associated suppression of tumor immunity, which appears to be induced by pro-inflammatory factors, might play a significant role in the development and progression of CRC. Therefore, IDO in PGE2/EP4 signaling appears to be one of the major targets of EP4 antagonist for suppressing colitis-related colorectal tumorigenesis.

Precise mechanisms of IDO suppression by RQ-15986 are unclear on AOM/DSS-induced CRC model. In this rodent model, pro-inflammatory cytokines, including IFN-γ, might be up-regulated in colonic mucosa by DSS administration. RQ-15986 attenuated the inflammation in colonic mucosa, which might lead to IDO suppression. In CRC tissues, IDO might be suppressed by RQ-15986 in the similar way as previously reported that inhibition of EP4 down-regulated IDO expression in cancer cells and monocyte-derived dendritic cells [12,13]. According to the previous reports [7,13], and the present study, both EP4 and IDO appear positive on the epithelial cells and tumor cells, and both EP4 and IDO are presumably thought to express in the same cells. Further studies are needed to investigate the relationship of these expressions and how they affect each other.

In order to assess effectiveness of chemopreventive agents against CRC, an appropriate animal model mimicking human colorectal carcinogenesis is needed. For this purpose, we employed *Apc*-mutant KAD rat which was developed by Yoshimi *et al* [35] and used in various cancer studies including CRC considering features of *Apc* mutation [36–38]. The AOM/DSS-induced KAD rat CRC model used in the present study is very reasonable because the model involves *Apc* mutation, exposure of carcinogen, and tissue inflammation, all of which are associated with human CRC development [39,40]. The usefulness of this model for the study of colorectal cancer chemoprevention is also recognized because it enables sequential observation of CRC development and growth and mucosal inflammation through endoscopic examination [19,20]. One limitation of employing KAD rat in the study of colitis-associated CRC (CAC) is the molecular mechanism of genetic mutation. CAC is related with mutation of the gene encoding p53 as an early event, which is different from sporadic colon tumors with APC mutation as well as tumors in KAD rats [41]. Therefore, CRC developed in KAD rats may be different from CAC and may not be suitable as the rodent model for inflammation-associated colon tumors. According to the previous report [35,40], KAD rats showed no spontaneous colon tumor and AOM-treated KAD rats did not develop any colon tumors. In addition, KAD rats were thought to be susceptible to colonic inflammation induced by DSS because AOM/DSS-treated control rats displayed significantly less colon tumors. These suggest that APC mutation alone may not be a direct factor for colon tumor development in AOM/DSS-treated KAD rats as molecular mechanism of genetic mutation. Since colon tumors in KAD rats appeared to be initiated by AOM and then promoted by DSS-induced colonic inflammation, those can be considered at least as inflammation-associated tumors.

It is not appropriate to discuss the effects of chemopreventive agents and anti-cancer agents in the same line; however, a highly selective antagonist of EP4 is expected to exert anti-tumor activity. For instance, E7046, a selective antagonist of EP4, demonstrated the best response of stable disease with manageable tolerability and immunomodulatory effects in a clinical trial that enrolled patients with advanced cancer [42]. Several studies also indicate that using EP4 antagonists, particularly in combination with either chemotherapy or immune-based therapies, is a promising novel approach to cancer therapy [43]. Therefore, the safety and usefulness of EP4 antagonists, including RQ-15986, as a chemopreventive agent, should be examined in more detail. This may lead to the clinical application of EP4 antagonists in the future.

In conclusion, RQ-15986, a selective EP4 antagonist, suppressed colon carcinogenesis presumably through attenuation of inflammation and regulation of the immune system in the colon. EP4 antagonists may be potential agents for preventing colitis-related CRC development.

## Supporting information

S1 Fig Histopathology in the liver, spleen, and kidney of the experimental mice. Representative photomicrographs of HE staining of liver, spleen, and kidney sections from the experimental mice. Bars, 200 μm.
(TIF)

S1 Table Primer sequences. (DOCX)
