# Supplementary figures and images for: Inhibitory effects of a selective prostaglandin E2 receptor antagonist RQ-15986 on inflammation-related colon tumorigenesis in APC-mutant rats
Source: PLoS One. 2021 May 18;16(5):e0251942. doi: 10.1371/journal.pone.0251942 (PMC8130959; doi:10.1371/journal.pone.0251942)

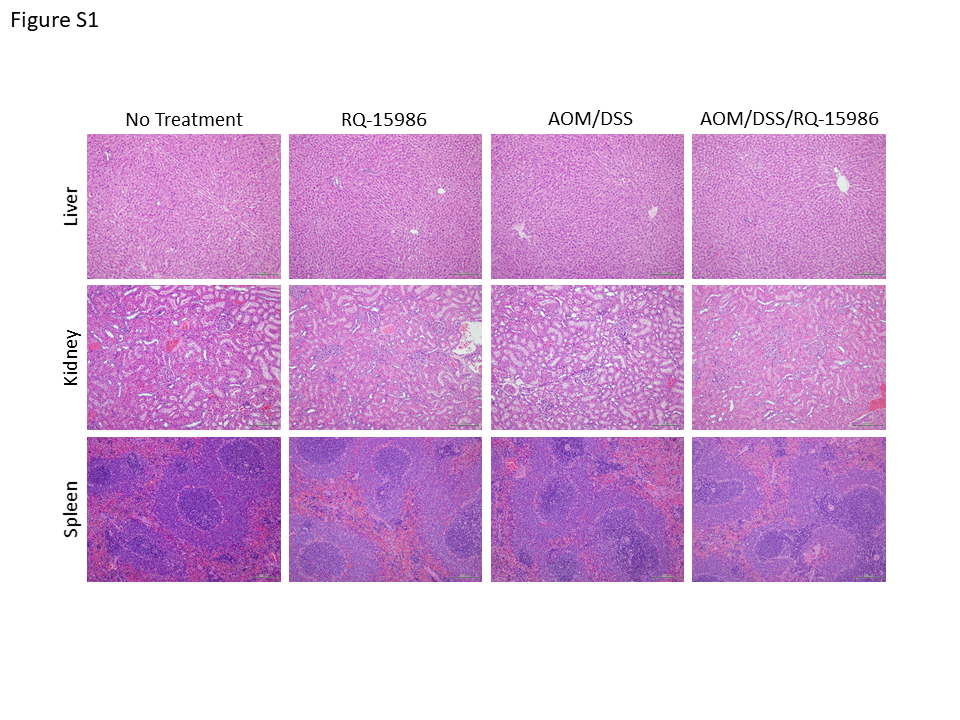

Supplement: S1 Fig — Representative photomicrographs of HE staining of liver, spleen, and kidney sections from the experimental mice. Bars, 200 μm. (TIF) [file pone.0251942.s001.TIF]
