# Supplementary material for: Inhibitory effects of a selective prostaglandin E2 receptor antagonist RQ-15986 on inflammation-related colon tumorigenesis in APC-mutant rats
Source: PLoS One. 2021 May 18;16(5):e0251942. doi: 10.1371/journal.pone.0251942 (PMC8130959; doi:10.1371/journal.pone.0251942)
Supplement: S1 Table — (DOCX) [file pone.0251942.s002.docx]

**Table S1. Primer sequences**

| Gene | Forward | Reverse |
| --- | --- | --- |
| (rat) |  |  |
| *Foxp3* | gtacagccggacacactgc | gctgacttccaagtctcgtgt |
| *Gapdh* | tgggaagctggtcatcaac | gcatcaccccatttgatgtt |
| *Ido1* | gggctttgctctaccacatc | tagccacaaggatccaaggt |
| *Ifng* | ttttgcagctctgcctcat | agcatccatgctacttgagttaaa |
| *Il6* | cccttcaggaacagctatgaa | acaacatcagtcccaagaagg |
| *Il18* | gcctgatatcgaccgaaca | ccttccatccttcacagatagg |
| *Mcp1* | cgtgctgtctcagccagat | ggatcatcttgccagtgaatg |
| *Tnfa* | agttggggagggagacctt | catccacccaaggatgtttag |
| (human) |  |  |
| *GAPDH* | agccacatcgctcagacac | gcccaatacgaccaaatcc |
| *IDO1* | gtgtttcaccaaatccacgat | ctgatagctgggggttgc |
